# Long-Range Diagnosis of and Support for Skin Conditions in Field Settings

**DOI:** 10.3390/tropicalmed3030084

**Published:** 2018-08-13

**Authors:** Victoria Williams, Carrie Kovarik

**Affiliations:** Department of Dermatology, Perelman School of Medicine, University of Pennsylvania, Philadelphia, PA 19104, USA; carrie.kovarik@uphs.upenn.edu

**Keywords:** teledermatology, eHealth, mHealth, long range diagnosis, dermatology, telepathology, technology, skin disease

## Abstract

Skin diseases are a significant cause of morbidity and mortality worldwide; however, access to dermatology services are critically limited, particularly in low- to middle-income countries (LMIC), where there is an overall shortage of physicians. Implementation of long-range technological support tools has been growing in an effort to provide quality dermatology care to even the most remote settings globally. eHealth strategies can provide realistic healthcare solutions if implemented in a feasible and sensitive way, customizing tools to address the unique needs and resource limitations of the local setting. This article summarizes the various types of telemedicine and mobile health (mHealth) tools and their practical applications and benefits for patient care. The challenges and barriers of teledermatology are discussed, as well as steps to consider when implementing a new teledermatology initiative. eHealth arguably offers one of the most flexible and realistic tools for providing critically needed access to dermatology skills in underserved LMICs.

## 1. Introduction

Skin diseases are a significant cause of morbidity and mortality worldwide; however, access to dermatology services are critically limited, particularly in low- to middle-income countries (LMICs), where there is an overall shortage of physicians [1]. The World Health Organization (WHO) has estimated that there is a worldwide shortage of 4.3 million physicians and nurses, and countries with the lowest density of healthcare workers such as sub-Saharan Africa, have the highest level of disease burden [2,3,4]. When an estimated 1 billion people across the world do not have access to a trained healthcare worker, access to specialty services like dermatology care is very rare [3,4]. Many regions completely lack a dermatology specialist or have dermatologists that live only in urban areas, leaving remote populations without access to care [5,6]. Skin diseases are reported to be the 4th leading cause of disability worldwide, but with the limited number and distribution of dermatologists, it is nearly impossible to provide adequate care to everyone in need using traditional methods [7]. Thus, the implementation of long-range technological support tools has been growing in an effort to provide quality dermatology care to even the most remote settings globally. eHealth strategies can provide realistic healthcare solutions if implemented in a feasible and sensitive way, customizing tools to address the unique needs and resource limitations of the local setting.

The WHO defines eHealth as the overall use of information and communication technology for health, which can broadly apply to all parts of the healthcare system from electronic medical record systems, education, research, clinical care, and hospital information systems [8]. There are numerous subsets of eHealth that can augment the delivery of healthcare; however, when looking to improve the diagnosis and management of skin diseases in remote field settings, mobile health (mHealth) and more specifically mobile telemedicine tools can provide flexible and innovative solutions.

Telemedicine is a subset of eHealth that encompasses the use of electronic communications technology to exchange medical information for the purposes of health and education. Dermatology is uniquely suited for telemedicine because of the largely visual component involved in diagnosis. Teledermatology can be delivered using either store and forward or live methods. Store and forward (SAF) methods, which are the most commonly utilized, involve gathering data that is then sent to a distant provider to be reviewed at a later time. Live telemedicine utilizes videoconference technology to connect a patient or provider in real time with a distant provider for consultation. Live telemedicine is infrequently used in the developing world due to the difficulty in sustaining a live connection.

Mobile health encompasses the use of mobile devices to support healthcare practices via various applications. The estimated penetration of unique mobile subscribers worldwide reached 66% (5.0 billion) in 2017, and is expected to be 71% (5.9 billion) by 2025 [9]. The majority of the world’s cell phone subscriptions are now in the developing world, and mobile phones offer an accessible healthcare tool that can be utilized even in the most remote settings. The type of healthcare that can be delivered through mHealth depends on the device and network connectivity. In 2017, smartphones as a percentage of mobile phone penetration were 34% in sub-Saharan Africa, and are projected to be 68% in 2025. Including 3G, mobile broadband network coverage reached 83% globally in 2016 [9]. As the connectivity advances in LMIC, the opportunities for mHealth expand.

## 2. Types of Mobile Health (mHealth) and Telemedicine Tools

eHealth tools come in a variety of formats that may be customized depending on the needs of the users. Table 1 outlines the various platforms that can be leveraged to practice teledermatology and summarizes their associated advantages and disadvantages. Formal telemedicine platforms utilize programs that have been specially designed to transmit secure healthcare information between providers. Traditionally, SAF teledermatology platforms were created solely for desktop use and required uploading photographs to a web-based program, which could prove challenging for providers with low computer literacy, limited internet access, and poor computer resources. In recent years, most web-based teledermatology platforms have also developed an associated mobile application that functions in parallel to the web-based program and allows for easier data collection and flexible connectivity over mobile networks or wifi. Providers can work directly from their mobile smartphones to transmit photos and patient information to remote consultants.

**Figure 1:**
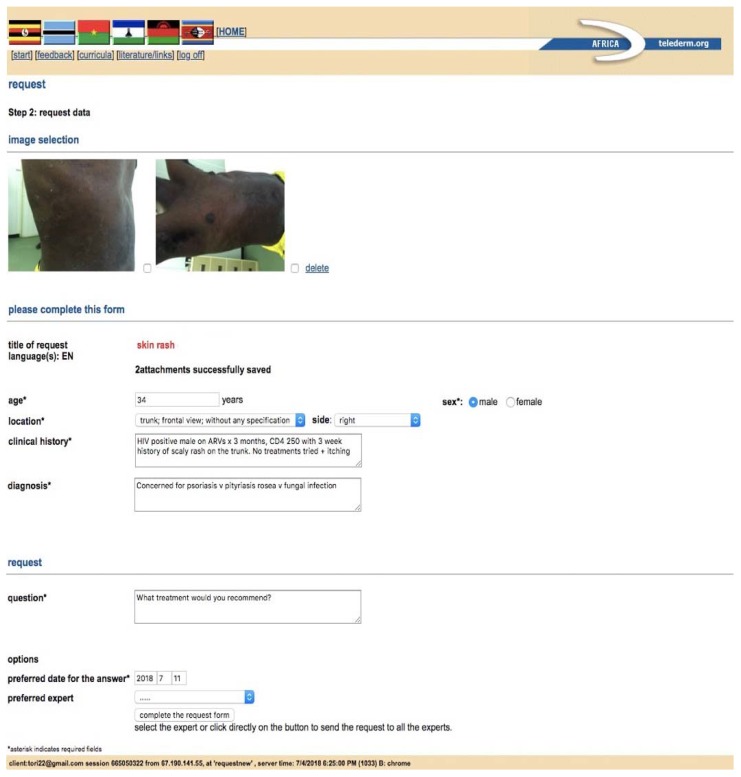
An example of a formal web-based teledermatology platform, the Africa Teledermatology Project, which uses a simple template to collect patient data for consultations.

Formal SAF teledermatology platforms require each user to be registered in the program and are tailored to collect a predetermined set of information. A customized dermatology template can be provided for patient data collection, which allows providers with limited dermatology knowledge to perform a thorough skin history without significant prior dermatology experience. Photos of the patient can then be attached to the consultation (Figure 1). Benefits of this type of service include secure data transmission and higher likelihood of pertinent patient information being included in consultations. Limitations include the need to individually register users and train them to use the application. Most programs will work over a mobile connection but require a strong network signal for ideal performance. Examples of successful long-term teledermatology programs using formal SAF platforms to support developing countries include the Africa Teledermatology Project [10], the Swinfen Charitable Trust [11,12], the Médecins Sans Frontières Telemedicine Network [13] and Réseau Afrique Francophone de Télémédecine (RAFT) project [14].

Informal telemedicine platforms include any method that allows the electronic SAF transmission of patient data. These tools allow for the quick and easy exchange of data without the need to individually identify, register and train all users. However, they have the drawback of not providing any formal framework for the collection or organization of data. The consultation information provided may be missing key elements and the security of transmitted information cannot be guaranteed.

Email is one of the more commonly utilized informal teledermatology platforms. Photos and pertinent patient information are included in an email message which can be sent to one provider or a group of providers for consultation. For the patient data to be secure, the sender and each recipient included must utilize a secure email service. Academic institutions with teledermatology links to developing countries via email platforms include Emory University to a teaching hospital in Kabul, Afghanistan [43]; the Medical College of Wisconsin to Hillside Healthcare International in Belize [44]; and the University of Basel, which is linked to several remote clinical sites globally [45].

**Figure 2:**
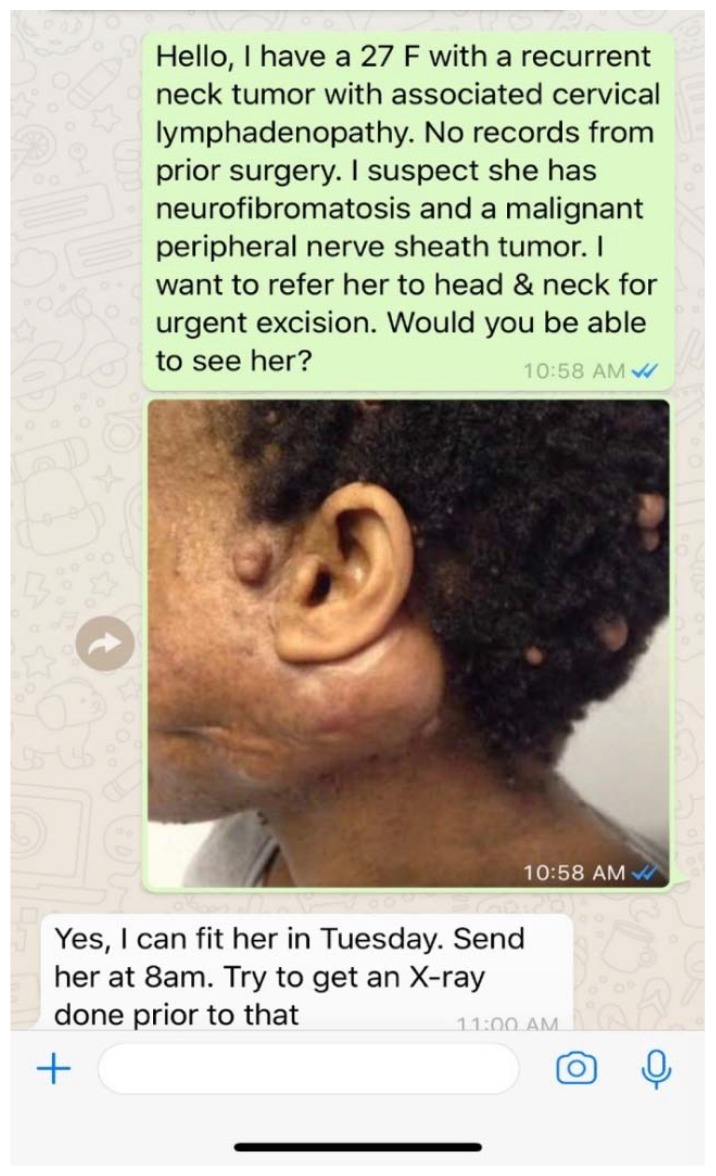
An example of secure direct messaging being used as a platform for teledermatology. Whatsapp is being utilized to tele-triage and coordinate care for a dermatology patient to sub-specialty care in Botswana.

Secure mobile messaging services allow the exchange of text, photos, audio, video and document files using mobile data or wifi. A widely used free mobile instant messaging application, Whatsapp, has been reported as a particularly powerful tool for teledermatology in several resource limited settings [23,24]. Because Whatsapp is already in use by more than 1 billion people in over 180 countries worldwide [46], the application can integrate seamlessly into the daily clinic routine of providers with minimal additional effort. Whatsapp can facilitate a variety of rapid patient care communications, allowing consulting physicians to learn about their patients and provide appropriate care in real time (Figure 2). Whatsapp does not require additional investments in equipment or dedicated internet access for users because it can be utilized on a provider’s own smartphone with either mobile data or wifi. Most importantly, Whatsapp maintains high functionality even in areas of poor connectivity. There are several other secure mobile messaging systems that have been designed for healthcare; however, most require paid subscriptions which limit their usage in LMICs [25,26,27,28,29,30,31,47].

Teledermatology can also be leveraged using cloud based file-hosting services that can provide a secure method for online file storage and sharing. Dropbox and Google drive are two popular cloud platforms that can facilitate Health Insurance Portability and Accountability Act (HIPAA) compliant sharing of protected patient information if utilized appropriately [19,20,48]. A recent study in Egypt, which used Dropbox for teledermatology consultation, found it to be a reliable diagnostic method with high rates of patient satisfaction [49]. Although cloud-based applications can function over mobile networks, they typically require a strong signal or wifi for optimal performance.

There has been a steady rise in the use of social networking among physicians who are starting to utilize crowdsourcing as a tool for patient care. Telederm.org is a free web-based dermatology networking platform that was started in 2002 and now has more than 2000 users [32]. After registering and creating an online profile, users can submit questions or cases to discussion forums that are divided by topic. Sermo is a similar website that connects more than 800,000 physicians of different specialties across the world via anonymous user profiles to allow the discussion of cases and other healthcare related topics [33]. Facebook, which connects users via a public profile, now has the ability to host private groups that are reportedly secure and only accessible by invitation [34]. Providers from any setting can come together to form collaborative groups in order to share challenging cases and request input from other members. Facebook groups can be tailored to specific topics of interest such as dermatopathology, tropical medicine or skin of color. Garcia-Romero et al. reported on the use of Facebook to create a teledermatology link between a rural clinic and a dermatology department at an urban general hospital in Mexico, which achieved clinical improvement in 75% of patients who received remote consultation [50]. Social media platforms have the benefit of a simple interface that requires negligible training for users and, most importantly, allows remote providers to connect with potentially thousands of dermatologists and other physicians across the world. Bandwidth requirements are low for these applications so they can be easily utilized on mobile devices in low-connectivity areas. However, there has been significant controversy about the use of social media platforms for healthcare, mainly due to the difficulty in guaranteeing patient privacy, confidentiality and security of the exchanged data. These methods should be used with caution.

Telepathology is a powerful component of teledermatology because histological analysis is vital for diagnostic confirmation in many dermatologic conditions, particularly in areas with high HIV burdens where clinical presentations are often atypical. Reliable pathology consultation services are critically needed in developing countries. Half of all fellowship trained pathologists work in the US, serving less than 5% of the global population [51]. Access to pathologists with dermatopathology experience or dermatopathology specialization is even more difficult to find in the global setting, creating an additional layer of challenges for physicians caring for skin diseases in remote settings. 

Telepathology methods can also be tailored to the needs of the local community and available resources. The three methods of telepathology currently described are static imaging, dynamic imaging, and virtual slide systems (Table 1).

**Figure 3:**
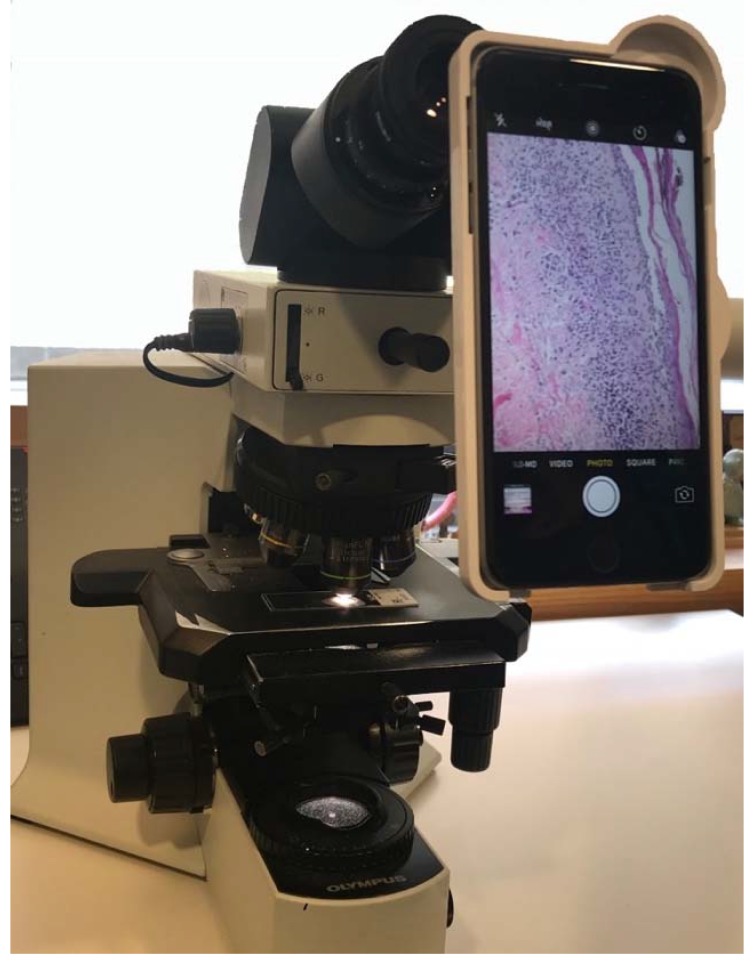
An example of a low-cost method for telepathology, the LabCam smartphone attachment, which allows photography of slides through the eyepiece of any microscope using an iPhone.

Static imaging is arguably the simplest telepathology method with the lowest budget requirements. The process involves taking photographs of slides at different magnifications and transmitting them to a remote dermatopathologist via any secure messaging, email or file-sharing platform. The most basic static technique utilizes a camera or smartphone to photograph a slide directly through a microscope eyepiece. Specialized adaptors and eyepiece attachments (Figure 3) have also been developed for use in combination with a smartphone to simplify the process of image capturing and improve the quality of images [52]. However, Bellina and Missoni demonstrated that quality images can be produced using any type of camera-equipped smartphone without the need for adaptors or additional technology [53], making this method ideal for low-resource settings. Digital camera attachments for microscopes are widely available and offer another simple mechanism for photographing slides. Multiple remote settings have achieved successful implementation of static telepathology programs with reported diagnostic concordance rates of up to 90.2% [45,54,55]. Major advantages of static image telepathology include minimal investment needed in equipment, training and maintenance, as well as the ability to function with unstable internet access. Static methods have the disadvantage of lower image quality, risk of sampling error and higher time requirements due to the need to select representative fields of view and take numerous pictures at different magnifications [56].

In dynamic imaging systems, slide images are examined in real time using a live microcopy viewing platform. Control of the live streaming images is either done directly by on site personnel or by the remote pathologist via robotic control of the microscope.

Live viewing systems have the advantage of allowing the pathologist to view the entire slide including different focus planes. Live view also allows cases to be discussed in real time which can increase the educational quality of consultations [57,58]. The feasibility of implementing a dynamic system has been demonstrated by the long-term use of a robotic telepathology system in a resource-limited government hospital in Botswana [59].

Virtual slide microscopy, also known as whole slide imaging systems, creates high-resolution scanned images of histology slides that can be digitally stored and then reviewed by a remote pathologist using specialized virtual slide viewing software [60]. This is also referred to as a hybrid method because it allows a pathologist to analyze the entire histology slide image dynamically by viewing selected areas digitally at higher magnification [61]. Major advantages include the ability to automate slide scanning; reduce interpretation time compared to robotic or static methods; the ability to manipulate, annotate and analyze slide images with viewing software; and the numerous educational applications that can be generated by creating a virtual ‘teaching set’ [61,62].

Virtual and dynamic slide telepathology systems can offer visual quality that is comparable to viewing slides in person under light microscopy, and several studies support good diagnostic concordance compared to a traditional glass slide review [63,64,65,66,67,68,69,70].

However, commercial slide-imaging systems are difficult to implement in low-resource settings due to the high cost of hardware and software, the need for skilled technicians to operate and maintain the systems locally, and the need for consistent high-bandwidth connectivity to transmit quality images [56,60,71].

In an effort to overcome the cost barriers of commercial slide-imaging systems, several low-cost interventions have been piloted utilizing innovative telepathology methods. Dudas et al. tested three low-cost telecytology systems including a Raspberry Pi attached to a webcam, an iPhone 4S with FaceTime, and an iPhone 4S with a live-streaming application [72]. All systems were able to stream live video of cytology slides to remote locations at a resolution that was suitable for a pathologist’s review [72]. Meléndez-Álvarez et al. designed a telepathology prototype manufactured with plastic materials made using an open design 3D printer, a conventional optical microscope, a Celestron camera attachment, open-sourced software and electronic components that are readily available in most electronic shops [73]. The prototype had a total cost of less than $910 and the resulting images were judged to be of diagnostic quality by a pathologist [73]. Utilization of free web-based teleconferencing software such as Skype, Google Hangouts, GoToMeetings, Windows Live Messenger, Fuze, and Webex, can provide a low-cost alternative to commercial live view microscopy systems [60,74,75,76]. Yu et al. described the newly realized capacity of smartphones to power whole-slide imaging systems via software which splits the image digitalization process between smartphone applications and remote cloud servers [77]. An android or iOS smartphone mounted on the eyepiece of a standard optical microscope has the ability to scan whole-slide images into a virtual slide with a resulting image quality comparable to high end commercial slide-scanning systems [77,78]. This innovative smartphone technology offers the potential to bring slide scanning technology to more widespread settings in LMICs.

Implementation of any telepathology system requires careful planning and a strong partnership between local and remote providers. Additionally, it is important to note that none of these telepathology tools can overcome the need for local training and resources to perform quality skin biopsies and histopathological processing at the local site which is often the limiting factor in resource-limited settings [56]. Table 2 summarizes the advantages and disadvantages of each type of telepathology tool.

In addition to supporting teledermatology functions, eHealth tools can provide access to education in many forms. The RAFT network is an example of a robust telemedicine network spanning four continents that focuses on providing medical education through a variety of low-bandwidth technologies such as interactive video lectures, virtual patient encounters, continuing medical education curriculums, tele-consults, clinical decision support tools, and web-casting of scientific conferences [14,79]. The emphasis on local involvement in coordinating and creating educational content that is most relevant to local providers has helped to make this project sustainable and successful [79].

mHealth applications are powerful educational tools because they can provide vital information to clinicians at the point of care. In recent years the number of mHealth applications has rapidly expanded to include programs to assist with disease diagnosis, management guidelines, drug reference, evidence-based medicine search tools, medical calculators, medical training tools, and clinical decision support pathways for navigating difficult clinical scenarios [80]. DynaMed, Epocrates, Medscape, Visual Dx, and UpToDate are some of the more commonly used mHealth tools, which allow clinicians to have rapid access to reference material with only a smartphone and/or mobile connection [81,82,83,84,85]. However, some of these applications require subscription costs, which can limit widespread usage in LMICs.

## 3. Benefits and Practical Applications of eHealth Tools

eHealth tools can be harnessed in a variety of different formats and combinations to benefit all levels of providers. Table 2 summarizes various methods in which teledermatology can provide solutions for the challenges of dermatology care in remote settings.

Teledermatology methods have been utilized to extend dermatologic care to developing countries since the early 1990s. The benefits of utilizing eHealth technologies are many and reports of successful programs have been published from numerous centers in Africa, Asia, and Latin America [5,10,11,44,86,87,88,89]. The growing transition to mobile teledermatology applications is supported by reports of successful implementation including satisfactory diagnosis and management of skin disease, diagnostic concordance with traditional face to face visits, patient satisfaction and provider satisfaction [23,89,90,91,92,93,94,95,96]. Utilization of tele-triage has the potential to significantly decrease the time to first evaluation of a patient by a dermatology specialist, which can in turn decrease morbidity from skin disease and increase patient satisfaction [23,97]. mHealth tools can improve the dermatology skills of front line providers who are more likely to retain knowledge because they are discussing and learning from their own patients [94]. Resident trainees in Botswana who were provided with smartphones pre-loaded with clinical decision support applications and training on how to best utilize the applications, reported multiple benefits including increased collaboration between primary care physicians and trainees, as well as increased clinician empowerment [77]. Teledermatology opens new communication and collaboration possibilities for providers and patients in isolated rural settings which can increase job satisfaction and improve care for patients with skin disease. Teledermatology can reduce healthcare costs by reducing the travel burden for patients, improving long-term follow-up and increasing successful care collaboration to improve patient outcomes [98,99]. Importantly, several studies have shown there is diagnostic concordance between teledermatology consultation and face-to-face visits, ensuring that patients are benefitting from use of the telemedicine without sacrificing quality of care [88,89,91].

## 4. Challenges and Barriers to Use of Teledermatology

Teledermatology is not without its challenges. Principally, sustainability becomes a key concern when setting up a program. Formal teledermatology platforms in particular require a significant amount of funding to implement, including but not limited to creation of the application, providing the necessary equipment, and training local providers. If the resources are not in place to continue the program long term, it becomes difficult to rationalize the initiation. The computer literacy of the providers being supported, local power supply, internet access, local mobile network connectivity, and availability of local technological resources all need to be carefully considered [14]. A major key to sustainability is ensuring that local clinicians are supportive of the program. Effective training on the tools being implemented can be challenging due to the often rapid provider turnover in low-resource clinical settings. Programs have a better chance of long-term success if technologies that are already in place and regularly used by local providers can be harnessed for teledermatology. Integration of the teledermatology program into the local healthcare system is essential for sustainability. Ideally the dermatologists answering the consultations are located in the country of origin of the consultee, or at the minimum, they are highly familiar with the diseases, work flow, and resources of the local healthcare system. Treatment plans being recommended must be realistic within the local socio-cultural and healthcare environment. Ideally, consultants should be available for continued collaboration and follow-up to address the common challenges that come with dermatology care in resource-limited settings. However, many teledermatology applications lack a method for long-term follow-up. Additionally, in certain cultures, teledermatology may not be an acceptable form of healthcare because patients expect face to face care or are resistant to having their skin condition photographed. Although teledermatology and mHealth applications give providers access to dermatology expertise, they do not solve the inherent challenges of local resource limitations such as medication stock outs, lack of available specialists for referral and limited diagnostic testing services.

The use of teledermatology requires a critical sensitivity to patient privacy and data security. It can be challenging to ensure that all providers participating in teledermatology are obtaining patient consent to share their information, sharing it over a secure platform, and using de-identified information to maintain patient confidentiality. Social media-based platforms must be used with caution because security settings and data-sharing policies can change rapidly and affect the ethical use of the program for patient data.

## 5. Practical Tips for Implementation of a New Teledermatology Initiative

The most important part of developing a new teledermatology initiative is a thorough local needs assessment of the area to be supported [12,44]. The first step in this process is evaluating the local healthcare environment. Most importantly, do local providers want to engage in teledermatology? Is there a need for or an interest in improving dermatology skills? Who would need to approve the program? Until clear need and firm local support can be secured, it is not advisable to initiate a teledermatology collaboration.

Review the current methods and workflow being used for dermatology patients. Where is the closest dermatology provider? Who cares for dermatology patients in rural areas versus urban areas? How far do most patients have to travel to see a dermatologist? How does the local healthcare system work? What is the cost of care for patients?

Then, review the locally available technological resources. Do most providers and patients use mobile phones? How prevalent are smartphones? How widespread are cellphone networks and how strong/reliable is the signal in the areas being supported? Are computers and/or wifi available in the local clinics or hospitals being supported? How technologically literate are the providers and patients being supported?

Next, identify partners that will participate in the initiative. Clearly outline the requirements and duties of all parties to be involved from the beginning to prevent problems developing later on. Have a discussion with local providers to get a firsthand understanding of their biggest concerns and the major challenges they face in caring for dermatology patients. Present your ideas for teledermatology solutions and get local feedback on the feasibility and interest in the proposed platforms. Ensure the platform can maintain patient privacy and provide a secure exchange of information.

Then, utilize the information gathered to narrow down the best options for a teledermatology platform. Identify the costs required to initiate and sustain the proposed platforms. Identify funding sources that can meet the needs of your proposal. Organize an implementation team to train all providers involved on both sides. Most importantly, identify a local means of providing ongoing training and support for the program to ensure sustainability.

If funding and resources appear to be major limitations, but local providers and leadership are supportive of developing a collaboration, consider starting with a free secure messaging application like Whatsapp. It can be integrated into even the most limited resource settings with little need for training and a low burden of time and effort for local providers to implement. If there is a robust interest in teledermatology at the local level, a strong health infrastructure that includes a dermatologist, and funding available for supplying and maintaining technological tools, a more formal teledermatology program could be feasible.

For overall success, teledermatology partnerships need to benefit both sides and need to be set up with the clear involvement of local providers. Remote dermatologists need to be willing and able to offer realistic diagnosis and treatment advice by taking into consideration local cultural norms and resource limitations. This is made possible by creating an open dialogue with local providers for exchange of information to allow both sides to learn from the interaction and improve care over time.

## 6. Conclusions

eHealth arguably offers one of the most flexible and realistic tools for providing critically needed access to dermatology skills in underserved LMICs. eHealth resources are ideal because they offer a wide range of customizable tools to match the needs and available resources of local providers in any setting. As the cost of mobile technology continues to decrease, the opportunities for low-cost teledermatology applications will continue to increase, making it feasible for any provider in any setting to be connected to a global community of dermatologists.

## Figures and Tables

**Table 1:** Description of teledermatology and telepathology platform types, advantages, disadvantages and examples.

Platform Type	Advantages	Disadvantages	Examples
**Formal Teledermatology Platforms**			
Web and/or Mobile Teledermatology Applications	secure, guides referring providers through a dermatology consult, stores a record of all consults, most applications can be used on desktop or mobile device	must identify and register all users, must train all users, cost associated with creation of application or subscription to use, most time intensive for providers to utilize, difficult for providers to ask follow up questions, usually no mechanism for long term follow-up of cases, requires wifi or strong network signal	Africa Teledermatology Project [10], Swinfen Charitable Trust [11,12], MSF [13], ClickMedix [15], Azova [16]
**Informal Teledermatology Platforms**			
Secure Email	can be used on desktop or mobile device, minimal training needed for users, minimally time intensive, fits into most providers daily routine, options for free access	security depends on email server, requires wifi or moderate network signal, no structure to guide consults, email accounts may have limited storage capacity, provider must identify and obtain emails of consultants to connect	free encrypted email services: Proton mail [17], Tutanota [18]
Secure Cloud Based File Sharing	can be used on desktop or mobile device, options for free access, provides a mechanism for organized storage of patient information, minimally time intensive	limited storage on free versions, requires some training for users, users must register, requires wifi or strong network signal, moderately secure, no structure to guide consults	Dropbox [19], Google Drive [20], OneDrive [21], Box [22]
Secure Direct Messaging Applications	fits into providers daily routine, least time intensive, minimal training needed, allows real time communication during patient visits, options for text/photos/videos/audio messaging, allows open communication for follow up questions and patient follow up, options for free access, secure end to end encryption, allows one-on-one or group chats, works well with low signal or wifi	provider must identify a consultant and obtain a phone number to connect, no structure to guide consults, no organized record of consults or communications	Free: WhatsApp [23,24], MedTunnel [25],Bloomtext [26] Paid: Imprivata [27], TigerConnect [28], Voalte [29], QliqSoft [30], Spok Mobile [31]
Social Networking Sites	free, low time commitment, minimal training needed, allows connection to a single provider or a global network, any provider can register and connect, works well with low signal or wifi	cannot guarantee security, difficult to guarantee credentials and expertise of consultants providing advice, no structure to guide consults, no organized record of consults or communications	Telederm.org [32], Sermo [33], Facebook [34]
**Telepathology Platforms**			
Virtual Slide Microscopy (VSM)	secure, highest quality images, can view any part of the slide at any magnification, creates an organized library of cases for teaching or research, least time intensive for reviewer when slides are pre-scanned	high cost to purchase, ongoing costs to maintain equipment and software, requires significant training, needs high storage capacity for images, needs consistent and high bandwidth to function, slide scanning can be time intensive for sender	Olympus VS 120 [35], Zeiss Axio Scan.Z1 [36], Leica Aperio AT2 [37]
Dynamic Slide Microscopy (DSM)	secure, can view any part of the slide at any magnification, potentially lower cost to implement compared to VSM	highest bandwidth requirements which may limit image quality, requires strict program compatibility for viewing, requires significant training, ongoing costs to maintain equipment and software, most time intensive to use for sender and reviewer	Leica Aperio LV1 [38], 3dHistech Panoramic DESK II DW [39]
Static Imaging	lowest cost, works with any microscope, no software requirements, does not require consistent wifi	risk of sampling error, quality of images varies based on skills of photographer, can only view areas of tissue and magnification chosen by photographer, time intensive for sender and reviewer	smartphone to eyepiece attachments: LabCam [40], Magnifi [41], Snapzoom [42]; any smartphone camera through eyepiece; any digital microscope camera

Abbreviations: MSF = Médecins Sans Frontières.

**Table 2:** Summary of the methods in which teledermatology can be utilized in practice, associated benefits and recommended platforms for remote providers in field settings.

Applications of Teledermatology	Benefits	Recommended Platforms for Remote Providers
Tele-Triage	appropriate and timely scheduling of patients into dermatology clinic, timely referral of dermatology patients to other specialists	secure direct messaging
Primary Care to Dermatology Consultation	diagnostic and management support, building dermatology skills over time	secure direct messaging
Specialist to Dermatology Consultation	diagnostic and management support, care coordination, building dermatology skills over time	secure direct messaging
Dermatologist to Dermatologist Consultation	second opinion, subspecialty dermatologist consultation, super specialist consultation for rare diseases, decreases isolation and burnout	formal teledermatology application, secure email, secure direct messaging, cloud based file sharing
Telepathology	expert analysis of skin biopsy specimens, improved diagnostic accuracy of skin disease, training of local pathologists	static images via smartphone or digital microscope camera
Long Term Management	allows for provider to dermatologist follow up, allows patient to dermatologist follow up, improves patient compliance and patient outcomes	secure direct messaging, secure email, cloud based file sharing
Care Coordination	allows for group chats between various providers to save time and resources	secure direct messaging, secure email, cloud based file sharing
Dermatology Education	remote access to dermatology education in any setting, builds local capacity	web based learning modules, video lectures, virtual patient encounters, email or web based access to lectures/handouts/guidelines, clinical decision support tools
